# DMD Mask Construction to Suppress Blocky Structural Artifacts for Medium Wave Infrared Focal Plane Array-Based Compressive Imaging

**DOI:** 10.3390/s20030900

**Published:** 2020-02-07

**Authors:** Zimu Wu, Xia Wang

**Affiliations:** Key Laboratory of Optoelectronic Imaging Technology and System, Ministry of Education, School of Optoelectronics, Beijing Institute of Technology, Beijing 100081, China; wuzm1990@163.com

**Keywords:** DMD mask, blocky structural artifacts, aperture interference, focal plane array-based, compressive imaging, medium wave infrared

## Abstract

With medium wave infrared (MWIR) focal plane array-based (FPA) compressive imaging (CI), high-resolution images can be obtained with a low-resolution MWIR sensor. However, restricted by the size of digital micro-mirror devices (DMD), aperture interference is inevitable. According to the system model of FPA CI, aperture interference aggravates the blocky structural artifacts (BSA) in the reconstructed images, which reduces the image quality. In this paper, we propose a novel DMD mask design strategy, which can effectively suppress BSA and maximize the reconstruction efficiency. Compared with random binary codes, the storage space and computation cost can be significantly reduced. Based on the actual MWIR FPA CI system, we demonstrate the proposed DMD masks can effectively suppress the BSA in the reconstructed images. In addition, a new evaluation index, blocky root mean square error, is proposed to indicate the BSA in FPA CI.

## 1. Introduction

Medium wave infrared (MWIR), of which the spectrum region is between 3 and 5 μm, has many imaging advantages beyond visible spectrum, such as night time observation and penetrating fog imaging [1]. Depending on the thermal radiance emitted and reflected by the objects, MWIR imaging systems can create images [2]. Targets are usually highlighted in MWIR images, as a result of temperature, emissivity and reflectivity difference between targets and background. Unfortunately, the MWIR sensors in the imaging systems are typically of low-resolution [3]. In order to improve performance of target recognition and surveillance, MWIR sensors of high-resolution are desired, but they are usually greater than tens of thousands dollars [4]. Hence, in spite of the immense application potential, high-resolution MWIR sensors are beyond the reach of scholars and researchers in some research fields which could get the most benefit from the high-resolution MWIR sensors.

MWIR compressive imaging (CI) is an alternative method of obtaining high-resolution MWIR images with a low-resolution MWIR sensor. The CI theory states that high-fidelity images can be reconstructed, which have a larger number of pixels than that physically exist in the imaging sensor [5,6,7]. An extreme example of CI is a single pixel camera (SPC) [8,9,10], which can reconstruct images with several hundred thousand pixels with just one photodetector. The trade-off of SPC, of course, is that a plenty of compressed samples of the imaging scene have to be obtained. Each compressed sample corresponds to a linear projection of the whole imaging scene onto one intensity value from a set of known functions. Although SPC has demonstrated potential for several applications, including remote sensing [11], 3D imaging [12], and microscopy [13,14], it is essentially a highly sequential imaging system. The compressed sampling process can be speeded up by using a focal plane array-based (FPA) sensor instead of the photodetector in SPC. Although the FPA CI systems have been implemented in visible [15,16] and short-wave infrared (SWIR) wavebands [17], comparatively few systems are implemented in MWIR. To our knowledge, only two experimental studies in the area of MWIR FPA CI are reported: Mahalanobis of Lockheed Martin built the first MWIR FPA CI system [18], which is a “merged-type”, and we built a “separate-type” system [19].

In spite of the acceleration of imaging speed, FPA CI has brought new challenges to the quality of reconstructed high-resolution images. As observed by Chen [17], blocky structural artifacts (BSA) are present in the reconstructed images, because of slight numerical fluctuations among the compressed samples for the same block. For MWIR FPA CI, due to physical limitation of the size of digital micro-mirror devices (DMD), aperture interference phenomena would also be observed [19], which aggravate the BSA and declines the image quality. An elaborate design for a DMD mask could alleviate the aperture interference in the low-resolution MWIR images obtained by the FPA sensor. In the study by Mahalanobis of Lockheed Martin [18], random binary codes are used for DMD masks. This strategy of DMD mask design can avoid the serious BSA, however, at a high cost in terms of storage resources and computing time. In addition, for some blocks, the mask patterns may be the same in two or more DMD masks, which reduces the efficiency of image compressed sampling and image reconstruction. An alternative common strategy is using Hadamard matrix for DMD mask design [20,21,22,23]. Because of aperture interference, the direct application of Hadamard matrix for DMD masks leads to obvious variations of light and dark areas distribution across the low-resolution images. As a result, the quality of the reconstructed high-resolution MWIR images will decline with an increase in the number of the low-resolution images.

In our previous work [19], the Hadamard matrix is directly applied to the DMD mask design, where all blocks have the same pattern in each DMD mask. Due to the aperture interference phenomenon, BSA has been observed in reconstructed high-resolution MWIR images. In this work, we further explore the aperture interference phenomenon in the MWIR FPA CI system, and focus our attention on DMD masks design to suppress the BSA. According to the system model of FPA CI, the reasons why BSA exist in the FPA CI are discussed. Particularly, the relationship between aperture interference and BSA is highlighted for MWIR FPA CI. As a result of these observations, based on the Hadamard matrix, we have designed customized patterns of DMD masks. Compared with a simple application of Hadamard matrix, the proposed patterns can suppress BSA, at the cost of a little more storage space and computation cost. On the other side, storage space and computation cost can be significantly reduced by the proposed patterns than random binary codes. We have modified the smoothed projected Landweber (SPL) reconstruction algorithm proposed by Mun [24], making it compatible with the proposed DMD mask. In addition, aiming at the BSA in FPA CI, we have proposed a new evaluation index, blocky root mean square error (BRMSE), to indicate the reconstructed image quality.

The rest of this paper is organized as follows. In Section 2, we describe the actual MWIR FPA CI system and present the aperture interference phenomenon when the DMD masks are constructed directly from Hadamard matrix. Section 3 proposes the customized patterns of DMD masks, modifies the SPL algorithm and describes BRMSE in detail. In Section 4, based on experimental results obtained from actual MWIR FPA CI system, we show the proposed patterns of DMD masks can suppress aperture interference, and BRMSE objectively assesses BSA. Some conclusions are given in Section 5.

## 2. Aperture Interference in MWIR FPA CI

**Figure 1:**
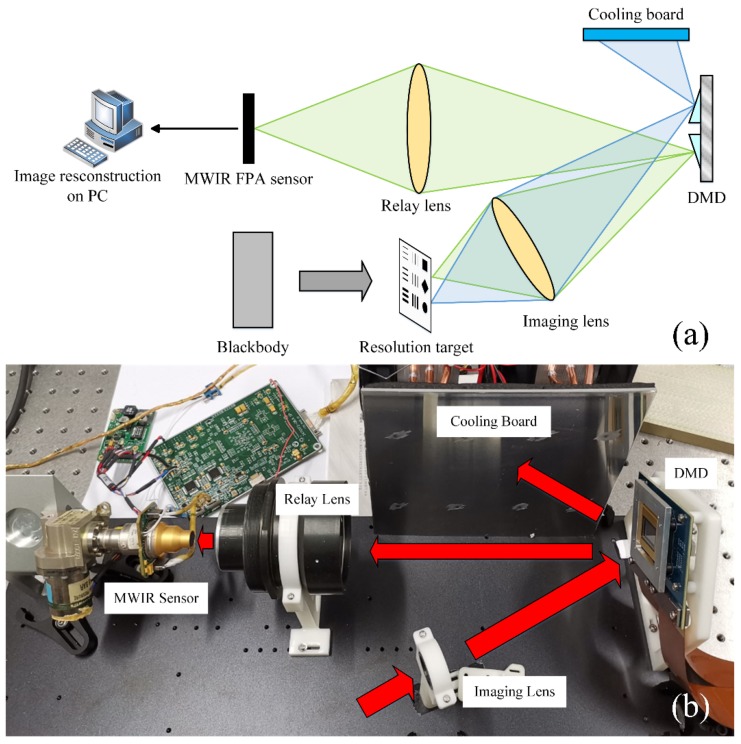
(**a**) Schematic diagram of our MWIR FPA CI system. The radiance of imaged scene is divided into two parts: one part to medium wave infrared (MWIR) sensor, and the other to cooling board, (**b**) Photograph of the actual system.

A cursory overview of our MWIR FPA CI system is necessary to define the issues and terms for the subsequent discussion, which has been described in detail in [19]. The schematic diagram and actual photo of our MWIR FPA CI system are depicted in Figure 1. The imaged scene is projected onto the DMD by the imaging lens. DMD (DLP9500, Texas Instruments, Dallas, TX, USA) is an array of numerous tiny mirrors and the resolution is 1920 × 1080. Each tiny mirror can be controlled individually, rotating to either +12° or −12° around the normal vector of DMD. The DMD mask patterns are implemented on the DMD loaded by control circuit. As each tiny mirror has two distinct rotation angles, the DMD reflect one part of the MWIR irradiation to the relay lens and the other part to the cooling board. The cooling board is placed at the symmetrical position to the imaging lens, along the optical axis of relay lens. The cooling board absorbs the radiant heat, reducing the environmental impact. Through relay lens, the MWIR irradiation of the imaged scene, the part reflected by DMD, is projected onto the MWIR sensor. Then, the irradiation is captured by a cooled MWIR sensor (LEO MW, Sofradir, Palaiseau, France), of which the resolution is 640 × 512, and the corresponding low-resolution images are generated. In the actual laboratory setup, the effective size of DMD and MWIR sensor is 1280 × 1024 and 320 × 256, respectively, realizing a strict correspondence between a block of 4 × 4 micro-mirrors in DMD and a single detector of the MWIR sensor. Finally, low-resolution MWIR images are sent to a computer for high-resolution image reconstruction. In our experiments, a plane blackbody (CDS 100-4, EOI, Goleta, CA, USA) is used as an MWIR radiation source, of which the infrared radiation intensity can be thought as approximately parallel and uniform. A transmission type of United States Air Force (USAF) resolution test chart can be placed in front of the blackbody, acting as the testing criteria for MWIR FPA CI.

In the MWIR FPA CI system, due to the material of the imaging lens and relay lens, the imaging waveband is 3.7~4.7 μm. Compared with the size of the micro-mirror, which is 10.8 μm × 10.8 μm, the MWIR wavelength is in the same order of magnitude. According to the practical rule-of-thumb [25], Fraunhofer diffraction will occur at an aperture with the greatest width b, if

(1) $$R>b^{2}/\lambda ,$$

where λ is the imaging wavelength. R is the smaller of the two distances, where one is from radiation source to the aperture, and the other is from the aperture to the imaging plane. Therefore, for MWIR FPA CI, the aperture interference is inevitable.

**Figure 2:**
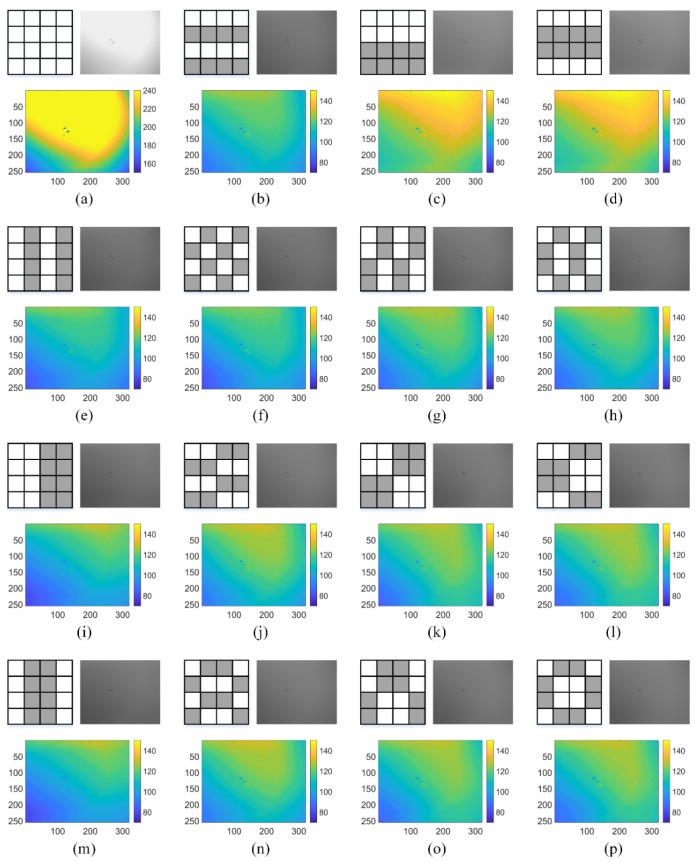
Aperture interference in MWIR FPA CI system with the direct application of Hadamard matrix. The patterns of DMD mask for each block and the corresponding low-resolution MWIR images are shown in (**a**–**p**), the color-mapped MWIR images are used to represent the distributions of light and dark areas. In the patterns, white square stands for “1” and grey square for “0”.

If the DMD masks are directly constructed from 16-order Hadamard matrix [19], namely, the pattern of each block is identical in each DMD mask and the patterns of all the masks are derived from the rows in a Hadamard matrix by fulling elements in column-first order, the low-resolution MWIR images have different light and dark distributions when different DMD masks are applied into the system. If a micro-mirror is placed at +12°, it is opened and indicated as “1”; otherwise, the micro-mirror is placed at −12°, it is closed and indicated as “0”. Using the blackbody as imaged scene, the low-resolution MWIR images have distinct gray-level distributions, as depicted in Figure 2. To highlight the gray level distributions of these images, different colors are used in the colormap. The gray level ranges are (150, 240) for Figure 2a and (70, 150) for Figure 2b–p. It is obvious that different DMD mask patterns lead to distinct distributions of light and dark areas in the MWIR images. It is noteworthy that the gray level distribution in each image is caused by two reasons: ${\left(\cos\right)}^{4}$ vignetting [26] and aperture interference. For the ${\left(\cos\right)}^{4}$ vignetting, as the “opened” micro-mirrors are not in parallel with the DMD plane, the brightest region is not located at the center of the MWIR image. The aperture interference changes the distributions of pixel gray values across the low-resolution MWIR images.

If the new coordinate systems, $X_{1}O_{1}Y_{1}$ and XOY, are established for DMD plane and detector plane, respectively, according to Fraunhofer diffraction [27], the radiance obtained by the MWIR sensor at the point (x,y) is

(2) $$E\left(x,y\right)=\frac{C}{f}\exp\left[ik\left(f+\frac{x^{2}+y^{2}}{2f}\right)\right]\iint_{\Sigma }\tilde{E}\left(x_{1},y_{1}\right)\exp\left[-i\frac{k}{f}\left(xx_{1}+yy_{1}\right)\right]dx_{1}dy_{1},$$

where C=1/iλ, k=2π/λ. f is the focal length of relay lens. $\tilde{E}\left(x_{1},y_{1}\right)$ is the radiance modulated by the DMD at $\left(x_{1},y_{1}\right)$. The radiance of $\left(x_{1},y_{1}\right)$ is equal to the imaging scene when the micro-mirror is ‘1’, or zero when the micro-mirror is ‘0’.

From Equation (2), we can clearly conclude that the aperture interference phenomenon in low-resolution MWIR images is closely related to the DMD masks. In particular, when the opened micro-mirrors are aligned into parallel lines, such as 2b, 2c, 2d, 2e, 2i and 2m in Figure 2, multi-slit interference occurs.

For FPA CI, the gray values of all pixels in the reconstructed high-resolution image are computed from the low-resolution MWIR images. A FPA CI system can be viewed as an array of SPCs [28,29,30], thus the system model can be expressed in a matrix notation as

(3) $$y_{i}=\Phi_{i}x_{i},$$

where, for the *i*th detector of MWIR FPA sensor, $y_{i}\in R^{m}$ is the set of m compressed samples, $x_{i}\in R^{n}$ represents the high-resolution image block, and $\Phi_{i}\in R^{m\times n}$ is the measurement matrix for the block.

In the practical computation, $y_{i}$ is consisted of the *i*th pixel of all low-resolution MWIR images, and each row of $\Phi_{i}$ is corresponding to the *i*th block pattern of the DMD mask. For example, if the DMD masks of Figure 2a–d are used for image reconstruction, Equation (3) can be rewritten as

(4) $$\left[\begin{array}{c} y_{i}^{\left(1\right)}\\ y_{i}^{\left(2\right)}\\ y_{i}^{\left(3\right)}\\ y_{i}^{\left(4\right)} \end{array}\right]=\left[\begin{array}{llllllllllllllll} 1 & 1 & 1 & 1 & 1 & 1 & 1 & 1 & 1 & 1 & 1 & 1 & 1 & 1 & 1 & 1\\ 1 & 0 & 1 & 0 & 1 & 0 & 1 & 0 & 1 & 0 & 1 & 0 & 1 & 0 & 1 & 0\\ 1 & 1 & 0 & 0 & 1 & 1 & 0 & 0 & 1 & 1 & 0 & 0 & 1 & 1 & 0 & 0\\ 1 & 0 & 0 & 1 & 1 & 0 & 0 & 1 & 1 & 0 & 0 & 1 & 1 & 0 & 0 & 1 \end{array}\right]\left[\begin{array}{c} x_{i,1}\\ x_{i,2}\\ \vdots \\ x_{i,16} \end{array}\right],$$

where $y_{i}={\left[y_{i}^{\left(1\right)},y_{i}^{\left(2\right)},y_{i}^{\left(3\right)},y_{i}^{\left(4\right)}\right]}^{T}$ and $x_{i}={\left[x_{i,1},x_{i,2},\cdots ,x_{i,16}\right]}^{T}$. In this work, to make the symbols clear and understandable, subscript is used to indicate the pixel position and superscript is for the number of the low-resolution MWIR image. The elements of measurement matrix and $x_{i}$ are consisted of DMD mask patterns and the *i*th high-resolution MWIR image block in column-first order, respectively.

Whatever image reconstruction algorithm is selected, the essence is solving the underdetermined equations, but the detailed process is different. If $y_{i,1}=2\times y_{i,2}=2\times y_{i,3}=2\times y_{i,4}$ is not satisfied, BSA will occur. For MWIR FPA CI, aperture interference brings a great gray value difference at the same pixel position when the number of opened micro-mirrors are identical, which can be observed in Figure 2b–p.

**Figure 3:**
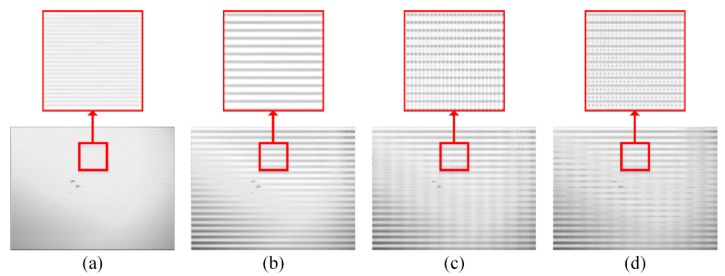
The high-resolution medium wave infrared (MWIR) images are reconstructed at (**a**) 0.125, (**b**) 0.25, (**c**) 0.375 and (**d**) 0.5. A small area of each image is zoomed in to highlight the blocky structural artefacts (BSA).

If high-resolution images are reconstructed from these low-resolution MWIR images, in which aperture interference phenomenon is obvious, the image quality is seriously influenced by BSA. In Figure 3, the reconstructed high-resolution images with compression ratios of 0.125, 0.25, 0.375 and 0.5 are shown, and the same areas are zoomed in to highlight the BSA.

## 3. Image Reconstruction and BRMSE

In traditional block-based compressed sensing algorithm, all blocks have the same pattern to simplify computation [24,31]. In our previous work [19], we applied this strategy to our MWIR FPA CI system, however, this led to serious aperture interference. Random binary codes for DMD masks could avoid the pattern repetition for all blocks [18], but tremendously increase storage burden and computing time. Moreover, in the strategy of random binary code, the patterns for the same block may be identical, which reduces the efficiency of compressive sampling as well. Thus, in this work, we also designed the DMD masks with the Hadamard matrix, but extended the circular pattern area, which slightly increased storage and computation expense, but remarkably suppressed the BSA in the reconstructed high-resolution images.

In this section, we first describe the generation process of the proposed DMD masks, which can suppress BSA in MWIR FPA CI. According to the characters of the proposed DMD masks, SPL [24] is also revised. Then, we discuss the storage space and computation cost of the proposed DMD mask design strategy. Depending on the theoretical analysis, the proposed DMD masks are proved to have dominant advantages over using random binary code. At last, based on the generation causes of the BSA in FPA CI, BRMSE is proposed to quantitatively evaluate the image quality of reconstructed high-resolution images.

### 3.1. DMD Masks

According to the basic theory of wave optics [32], similar DMD mask patterns lead to the similarity of aperture interference in the low-resolution MWIR images, which can also be observed in Figure 2. In Figure 2c,d, for the whole mask, the patterns are two-row horizontal stripes, but with one-row displacement. This trend can also be observed in the pairs of g-h, i-m, k-l and o-p. Based on this observation, we can conclude that, if the DMD masks for MWIR FPA CI have a similar pattern, the captured low-resolution MWIR images would have a similar gray level distribution.

Therefore, the philosophy of proposed DMD mask design strategy is: binding up a group of adjacent blocks to form a big block. For one DMD mask, all big blocks have the same pattern, but the blocks in each big block are distinct. In any pair of DMD masks, the patterns of the blocks are the same, but with the positional difference in big blocks. As a result, the proposed DMD mask patterns exhibit the similarity in the whole, but are diverse within the local area.

**Figure 4:**
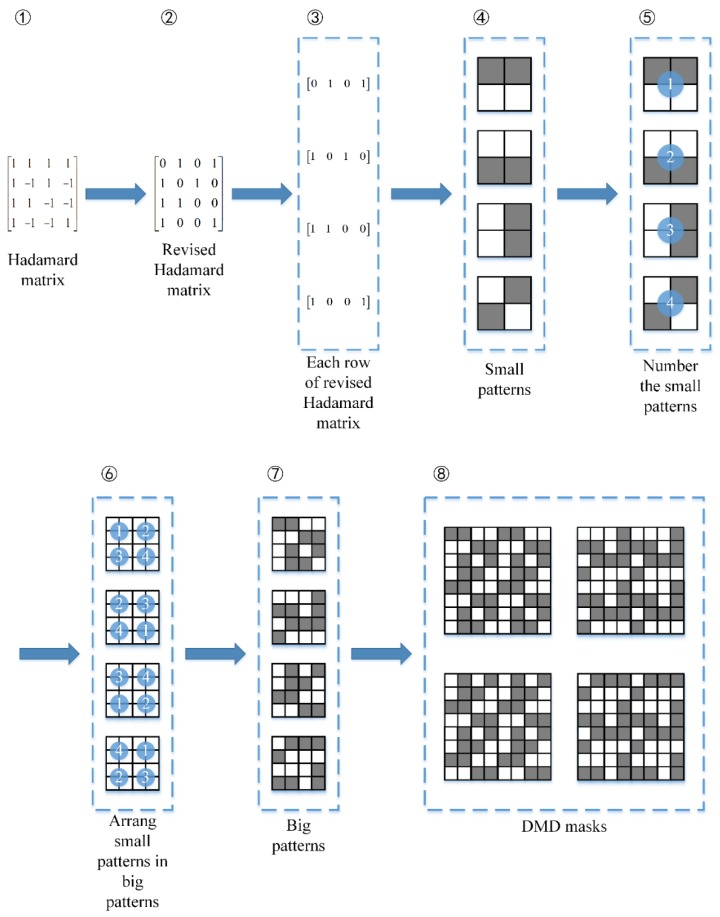
The process of DMD mask generation. For the purpose of simplification, the block size B is set to 4. In the patterns, white square stands for “1” and grey square for “0”.

Assume that in a MWIR FPA CI system, the block size is 2 × 2 micro-mirrors, and a big block consists of 2 × 2 blocks. The Hadamard matrix is used to construct the block pattern, which has two advantages in MWIR CI. Firstly, the Hadamard matrix is full rank, maximizing the compressive sampling efficiency. Secondly, in each block, half of the micro-mirrors are “open”, implicitly improving the signal-to-noise ratio of the MWIR image. The generation process of DMD masks involves eight steps: (1) Generate a Hadamard matrix with the order equaling to the big block size B, which is 4 for this system. (2) Revise the Hadamard matrix to applicable for DMD, turning all “−1” to “0”. Using the complement vector of the second instead of the original one, all blocks have the same number of opened micro-mirrors, which may reduce the impact of pixel crosstalk [33]. (3) Divide the revised Hadamard matrix into separate row vectors. (4) For each row vector, a small pattern for each block is generated by fulling the elements of the row in column-first order. (5) Number the small patterns, from 1 to B. (6) In a big block of the size B × B, number the blocks in a clockwise direction. (7) In big blocks, replace the numbered block with the small patterns to generate the big patterns. (8) Expand the big patterns to coverage the entire DMD area and the DMD masks are obtained. This generation process of DMD masks is illustrated in Figure 4.

### 3.2. Revised SPL Algorithm

In the work of Mun [24], the measurement matrix is orthonormal and identical for all blocks. However, for our MWIR FPA CI system and proposed DMD masks, the measurement matrices for the big block are non-normalized and discrepant. Thus, the SPL algorithm need to be revised for the MWIR FPA CI system, and the revised SPL algorithm are summarized in Algorithm 1.
**Algorithm 1** In the revised SPL algorithm, transform domain Ψ is chosen as dual-tree discrete wavelet transform (DDWT).1: **Input:** compressed samples Y, measurement matrix Φ, Ψ2: **Output:**
X3: **Initialize:**
$X^{\left(0\right)}=B\left(Y,\Phi \right)$, i=04: while $\left|D^{\left(i\right)}-D^{\left(i-1\right)}\right|\geq 10^{-4}$ do5:   i=i+16:   ${\overset{⌢}{X}}^{\left(i\right)}=\mathrm{Wiener}\left(X^{\left(i\right)}\right)$7:   ${\overset{⌢}{\overset{⌢}{X}}}^{\left(i\right)}=\overset{⌢}{B}\left({\overset{⌢}{X}}^{\left(i\right)},\Phi \right)$8:   ${\overset{⌣}{\overset{⌣}{X}}}^{\left(i\right)}=\Psi \cdot {\overset{⌢}{\overset{⌢}{X}}}^{\left(i\right)}$9:   ${\overset{⌣}{X}}^{\left(i\right)}=\mathrm{Threshold}\left({\overset{⌣}{\overset{⌣}{X}}}^{\left(i\right)},\lambda \right)$10:   ${\bar{X}}^{\left(i\right)}=\Psi^{-1}\cdot {\overset{⌣}{X}}^{\left(i\right)}$11:   $X^{\left(i+1\right)}=\bar{B}({\bar{X}}^{\left(i\right)},\Phi )$12:   $D^{\left(i\right)}=\frac{1}{\sqrt{N}}{\left\|X^{\left(i+1\right)}-{\overset{⌢}{\overset{⌢}{X}}}^{\left(i\right)}\right\|}_{2}$13: end while

In the revised SPL algorithm, $X^{\left(0\right)}$, ${\overset{⌢}{\overset{⌢}{X}}}_{j}^{\left(i\right)}$ and $X_{j}^{\left(i+1\right)}$ should be treated specially. For a block, its measurement matrix is determined by the position in the big block, so the functions of B(⋅,⋅), $\overset{⌢}{B}\left(\cdot ,\cdot \right)$ and $\bar{B}\left(\cdot ,\cdot \right)$ are closely related to block number j. Algorithm 2 shows the definition B(⋅,⋅), $\overset{⌢}{B}\left(\cdot ,\cdot \right)$ and $\bar{B}\left(\cdot ,\cdot \right)$.
**Algorithm 2** In the revised SPL algorithm, B(⋅,⋅), $\overset{⌢}{B}\left(\cdot ,\cdot \right)$ and $\bar{B}\left(\cdot ,\cdot \right)$ are all block-wise, thus they are bounded together to clarify.1: **Require:**
$Y=\left[y_{1},y_{2},\cdots ,y_{N}\right]$ and $X=\left[x_{1},x_{2},\cdots ,x_{N}\right]$ are block-wise2: **Require:** measurement matrix Φ for the first block in big block3: for block j=1 to N do4:   i=blockposition(j)−15:   $\Phi_{j}=\mathrm{circshiftup}(\Phi ,i)$6:   $B\left(Y,\Phi \right)\Rightarrow x_{j}^{\left(0\right)}=\Phi_{j}^{T}\cdot y_{j}$7:   $\overset{⌢}{B}\left({\overset{⌢}{X}}^{\left(i\right)},\Phi \right)\Rightarrow {\overset{⌢}{\overset{⌢}{x}}}_{j}^{\left(i\right)}={\overset{⌢}{x}}_{j}^{\left(i\right)}+\Phi_{j}^{T}\cdot {\left(\Phi_{j}\cdot \Phi_{j}^{T}\right)}^{-1}\cdot \left(y_{j}-\Phi_{j}\cdot {\overset{⌢}{x}}_{j}^{\left(i\right)}\right)$8:   $\bar{B}({\bar{X}}^{\left(i\right)},\Phi )\Rightarrow x_{j}^{\left(i+1\right)}={\bar{x}}_{j}^{\left(i\right)}+\Phi_{j}^{T}\cdot {\left(\Phi_{j}\cdot \Phi_{j}^{T}\right)}^{-1}\cdot \left(y_{j}-\Phi_{j}\cdot {\bar{x}}_{j}^{\left(i\right)}\right)$9: end for

Where the symbol ⇒ represents the function definitions for B(⋅,⋅), $\overset{⌢}{B}\left(\cdot ,\cdot \right)$ and $\bar{B}\left(\cdot ,\cdot \right)$ in the *j*th block. blockposition(⋅) returns the position of the *j*th block in a big block. In the proposed DMD masks, blocks in a big block are numbered in row-first order, and all big blocks are done in the same manner. circshiftup(Φ,i) circularly shifts the rows of Φ by i rows upward.

In addition, ${\left(\Phi \cdot \Phi^{T}\right)}^{-1}$ is added into the calculation of $\overset{⌢}{B}\left(\cdot ,\cdot \right)$ and $\bar{B}\left(\cdot ,\cdot \right)$, to eliminate the influence of the non-normalization of measurement matrix for the FPA CI system. If ${\left(\Phi \cdot \Phi^{T}\right)}^{-1}$ is not added, all of the image information would be lost.

### 3.3. Advantages of the Proposed DMD Masks

**Figure 5:**
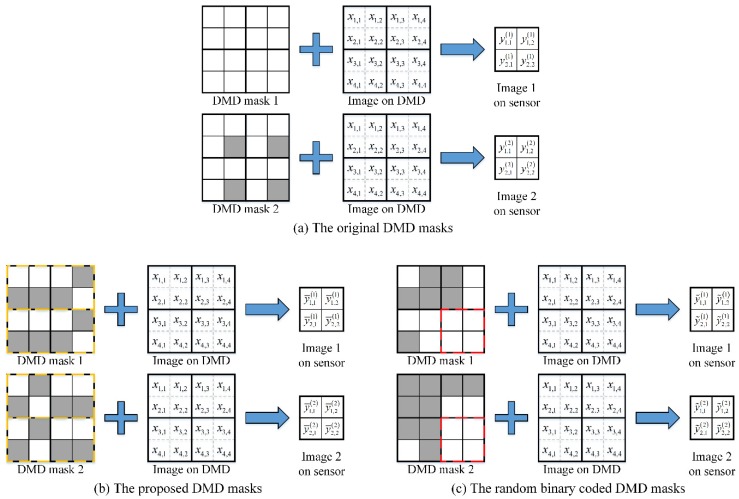
Imaging process of FPA CI. The images on DMD are the same in (**a**–**c**), but different DMD masks are used in the three imaging processes. The images on the sensor correspond to the low-resolution images, and the image on DMD is the high-resolution image, which is expected to be reconstructed.

To illustrate the difference of storage space and computation cost among the original DMD masks, the proposed ones and the random binary codes in FPA CI, we use the system models of the three to analyze the computing costs. The imaging process of FPA CI is shown in Figure 5. The image on DMD is the high-resolution image expected to be reconstructed, and the image on sensor is the low-resolution image. In FPA CI, the high-resolution image is computed from multiple times of low-resolution image sampling. To simplify the analysis, the resolutions of DMD and FPA sensor are set to 4 × 4 and 2 × 2 respectively. Figure 5 shows the twice low-resolution image sampling process with different strategies of DMD mask design.

In the original strategy of DMD mask design [19], as shown in Figure 5a, all blocks have the same pattern. According to Equation (3), for all pairs of DMD block and detector, the system model can be expressed as

(5) $$\{\begin{array}{c} \left[\begin{array}{c} y_{1,1}^{\left(1\right)}\\ y_{1,1}^{\left(2\right)} \end{array}\right]=\left[\begin{array}{llll} 1 & 1 & 1 & 1\\ 1 & 1 & 1 & 0 \end{array}\right]\left[\begin{array}{c} x_{1,1}\\ x_{2,1}\\ x_{1,2}\\ x_{2,2} \end{array}\right]\\ \left[\begin{array}{c} y_{2,1}^{\left(1\right)}\\ y_{2,1}^{\left(2\right)} \end{array}\right]=\left[\begin{array}{llll} 1 & 1 & 1 & 1\\ 1 & 1 & 1 & 0 \end{array}\right]\left[\begin{array}{c} x_{3,1}\\ x_{4,1}\\ x_{3,2}\\ x_{4,2} \end{array}\right]\\ \left[\begin{array}{c} y_{1,2}^{\left(1\right)}\\ y_{1,2}^{\left(2\right)} \end{array}\right]=\left[\begin{array}{llll} 1 & 1 & 1 & 1\\ 1 & 1 & 1 & 0 \end{array}\right]\left[\begin{array}{c} x_{1,3}\\ x_{2,3}\\ x_{1,4}\\ x_{2,4} \end{array}\right]\\ \left[\begin{array}{c} y_{2,2}^{\left(1\right)}\\ y_{2,2}^{\left(2\right)} \end{array}\right]=\left[\begin{array}{llll} 1 & 1 & 1 & 1\\ 1 & 1 & 1 & 0 \end{array}\right]\left[\begin{array}{c} x_{3,3}\\ x_{4,3}\\ x_{3,4}\\ x_{4,4} \end{array}\right] \end{array},$$

As the measurement matrices are same, Equation (5) can be integrated into a single expression, such as

(6) $$\left[\begin{array}{cccc} \begin{array}{c} y_{1,1}^{\left(1\right)}\\ y_{1,1}^{\left(2\right)} \end{array} & \begin{array}{c} y_{2,1}^{\left(1\right)}\\ y_{2,1}^{\left(2\right)} \end{array} & \begin{array}{c} y_{1,2}^{\left(1\right)}\\ y_{1,2}^{\left(2\right)} \end{array} & \begin{array}{c} y_{2,2}^{\left(1\right)}\\ y_{2,2}^{\left(2\right)} \end{array} \end{array}\right]=\left[\begin{array}{llll} 1 & 1 & 1 & 1\\ 1 & 1 & 1 & 0 \end{array}\right]\left[\begin{array}{cccc} \begin{array}{c} x_{1,1}\\ x_{2,1}\\ x_{1,2}\\ x_{2,2} \end{array} & \begin{array}{c} x_{3,1}\\ x_{4,1}\\ x_{3,2}\\ x_{4,2} \end{array} & \begin{array}{c} x_{1,3}\\ x_{2,3}\\ x_{1,4}\\ x_{2,4} \end{array} & \begin{array}{c} x_{3,3}\\ x_{4,3}\\ x_{3,4}\\ x_{4,4} \end{array} \end{array}\right],$$

Analogously, in the proposed strategy of DMD mask design, a big block consists of 1 × 2 blocks, and all big blocks have the same pattern, which is drawn in yellow dashed lines, as shown in Figure 5b. The system model can be expressed as

(7) $$\{\begin{array}{c} \left[\begin{array}{cc} \begin{array}{c} {\bar{y}}_{1,1}^{\left(1\right)}\\ {\bar{y}}_{1,1}^{\left(2\right)} \end{array} & \begin{array}{c} {\bar{y}}_{2,1}^{\left(1\right)}\\ {\bar{y}}_{2,1}^{\left(2\right)} \end{array} \end{array}\right]=\left[\begin{array}{llll} 1 & 0 & 1 & 0\\ 1 & 0 & 0 & 1 \end{array}\right]\left[\begin{array}{cc} \begin{array}{c} x_{1,1}\\ x_{2,1}\\ x_{1,2}\\ x_{2,2} \end{array} & \begin{array}{c} x_{3,1}\\ x_{4,1}\\ x_{3,2}\\ x_{4,2} \end{array} \end{array}\right]\\ \left[\begin{array}{cc} \begin{array}{c} {\bar{y}}_{1,2}^{\left(1\right)}\\ {\bar{y}}_{1,2}^{\left(2\right)} \end{array} & \begin{array}{c} {\bar{y}}_{2,2}^{\left(1\right)}\\ {\bar{y}}_{2,2}^{\left(2\right)} \end{array} \end{array}\right]=\left[\begin{array}{c} \begin{array}{cccc} 1 & 0 & 0 & 1 \end{array}\\ \begin{array}{cccc} 1 & 0 & 1 & 0 \end{array} \end{array}\right]\left[\begin{array}{cc} \begin{array}{c} x_{1,3}\\ x_{2,3}\\ x_{1,4}\\ x_{2,4} \end{array} & \begin{array}{c} x_{3,3}\\ x_{4,3}\\ x_{3,4}\\ x_{4,4} \end{array} \end{array}\right] \end{array},$$

In Equation (7), two expressions are used for the system model, because the big block consists of two blocks. For the blocks in one big block, the measurement matrices are distinct, so they should be treated separately. However, for the blocks at the same position of all big blocks, the measurement matrices are the same, and they can be integrated into one expression. This behavior is also reflected in Equation (7).

In the strategy of random binary codes, as the measurement matrices are distinct for all blocks, as shown in Figure 5c, all pairs of DMD blocks and detectors must be treated separately, and the system model can be expressed as

(8) $$\{\begin{array}{c} \left[\begin{array}{c} {\tilde{y}}_{1,1}^{\left(1\right)}\\ {\tilde{y}}_{1,1}^{\left(2\right)} \end{array}\right]=\left[\begin{array}{llll} 1 & 0 & 0 & 0\\ 0 & 0 & 0 & 0 \end{array}\right]\left[\begin{array}{c} x_{1,1}\\ x_{2,1}\\ x_{1,2}\\ x_{2,2} \end{array}\right]\\ \left[\begin{array}{c} {\tilde{y}}_{2,1}^{\left(1\right)}\\ {\tilde{y}}_{2,1}^{\left(2\right)} \end{array}\right]=\left[\begin{array}{llll} 1 & 0 & 1 & 1\\ 0 & 1 & 0 & 0 \end{array}\right]\left[\begin{array}{c} x_{3,1}\\ x_{4,1}\\ x_{3,2}\\ x_{4,2} \end{array}\right]\\ \left[\begin{array}{c} {\tilde{y}}_{1,2}^{\left(1\right)}\\ {\tilde{y}}_{1,2}^{\left(2\right)} \end{array}\right]=\left[\begin{array}{llll} 0 & 0 & 1 & 1\\ 0 & 1 & 0 & 1 \end{array}\right]\left[\begin{array}{c} x_{1,3}\\ x_{2,3}\\ x_{1,4}\\ x_{2,4} \end{array}\right]\\ \left[\begin{array}{c} {\tilde{y}}_{2,2}^{\left(1\right)}\\ {\tilde{y}}_{2,2}^{\left(2\right)} \end{array}\right]=\left[\begin{array}{llll} 1 & 1 & 1 & 1\\ 1 & 1 & 1 & 1 \end{array}\right]\left[\begin{array}{c} x_{3,3}\\ x_{4,3}\\ x_{3,4}\\ x_{4,4} \end{array}\right] \end{array},$$

It is noteworthy that, for the last expression of Equation (8), the rows of the measurement matrix are the same. As shown in Figure 5c, in the twice low-resolution image sampling, the DMD masks are identical for one block, which is drawn in red dashed lines. Theoretically, ${\tilde{y}}_{2,2}^{\left(1\right)}$ is equal to ${\tilde{y}}_{2,2}^{\left(2\right)}$, which means only one sampling is efficient for this block. The repeating pattern for some blocks, which is unavoidable for random binary codes, reduces the efficiency of image compressed sampling and image reconstruction.

In the FPA CI, the computing costs of image reconstruction is closely related to the number of matrix computation. In parallel computation, one thread is required for one matrix computation, and too many threads would dramatically increase computation burden, which is also a nightmare for software developers [34]. For the original strategy, only one matrix computation is needed. For the proposed strategy, the number of matrix computation equals to the big block size. However, for the random binary codes, the number of matrix computation equals to the number of detectors on the FPA sensor.

For larger-area FPA sensor, the gaps increases for the number of matrix computation among the three strategies. For our MWIR FPA CI system, the block size is 4 × 4 micro-mirrors, and a big block consists of 4 × 4 blocks. Thus, the number of matrix computation is 16 for the proposed strategy; that is 81,920 for random binary codes. In parallel computation, the number of threads is 1 for the original strategy, 16 for the proposed strategy, and 81,920 for random binary codes. In today’s multicore processors, 16 threads can be easily obtained, while 81,920 is far beyond reach. It is evident, compared with random binary codes, that the proposed strategy has an overwhelming advantage for computation cost. In addition, less expressions in the system model means less measurement matrices needed be stored in the computer memory; thus, storage space can also be saved in the proposed strategy to a greater extent than in using random binary codes. Consequently, based on the consideration of the algorithm complexity and real-time property of the MWIR FPA CI system, random binary codes may only be a good idea on paper.

### 3.4. BRMSE

In FPA CI, the BSA appear in the reconstructed high-resolution images, which reduces the image quality. Because of slight fluctuations in gray-level values of the same pixel among the low-resolution images, BSA is inevitable [17]. Aperture Interference, observed in MWIR, would aggravate the BSA. In the field of image sparse representation, the quality of the reconstructed image is generally evaluated by peak signal-to-noise ratio (PSNR). However, in FPA CI, the original high-resolution image is unobtainable, so PSNR cannot be used.

From the system perspective, each block can be treated independently, so BSA can be evaluated locally in every block. BRMSE is the mean value of the root mean square errors (RMSE) for all blocks, which can be expressed as:

(9) $$BRMSE=\frac{1}{N}\sum_{i=1}^{N}RMSE(block_{i})$$

where N is the number of blocks in one reconstructed high-resolution image, RMSE(⋅) calculates the RMSE of each block, which is defined as

(10) $$RMSE(block)=\sqrt{\frac{1}{n}\sum_{i=1}^{n}{\left(g_{i}-\bar{g}\right)}^{2}}$$

where RMSE(block) is the RMSE of the block, block, which has n pixels. $g_{i}$ is the gray value of the *i*th pixel and $\bar{g}$ the average gray value of block.

In order to evaluate BSA exactly, a uniform imaging scene—as in those of Figure 2 and Figure 3—is preferred for the calculation of BRMSE. Obviously, reconstructed images with less BSA have smaller BRMSE.

## 4. Experiment and Discussion

**Figure 6:**
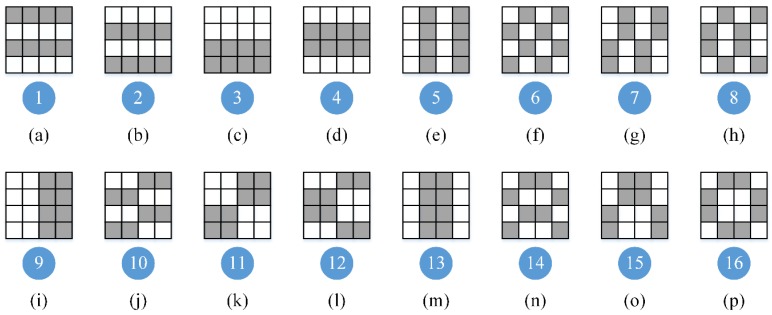
The numbers of the patterns for all blocks in DMD masks. The patterns of all blocks are generated from a 16-order Hadamard matrix. In (**a**–**p**), the block patterns are shown in the first row, and the numbers are depicted below the corresponding block patterns.

**Figure 7:**
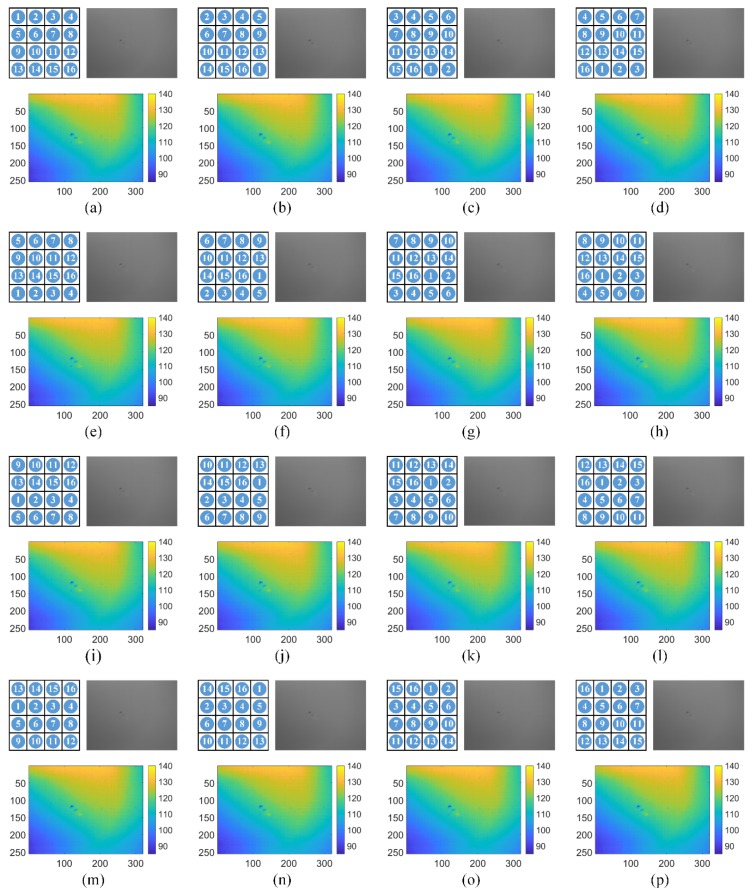
The low-resolution MWIR images obtained from the actual MWIR FPA CI system. In (**a**–**p**), each subfigure corresponds to one proposed DMD mask. In the first row, the patterns of the big blocks are depicted on the left, and the captured MWIR images are shown on the right. The color-mapped MWIR images in the second row are used to represent the distributions of light and dark areas. The imaged scene is a uniform blackbody.

For our MWIR FPA CI system, the block size is 4 × 4 micro-mirrors, and a big block consists of 4 × 4 blocks. According to the proposed strategy of DMD mask design, a 16-order Hadamard matrix is used to generate patterns for all the blocks, and the numbers of the patterns are depicted in Figure 6. Big blocks are consisted of these blocks, and a set of low-resolution MWIR images are obtained from the actual MWIR FPA CI system, as shown in Figure 7. The big block patterns are shown on the left in the first row, of which the size is 4 × 4 blocks. To highlight the gray level distributions of these images, color-mapped images are placed in the second row, and the gray level ranges are (85, 140). With the blackbody used as imaged scene, the images have almost identical light and dark distributions, which is quite different from that in Figure 2. Therefore, compared with a simple application of the Hadamard matrix, our proposed DMD masks can effectively suppress BSA in MWIR FPA CI, visually.

**Figure 8:**
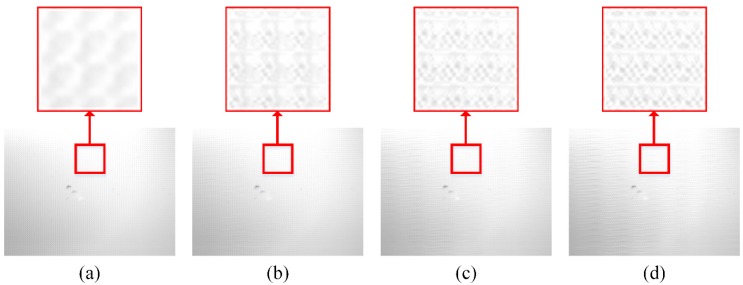
The high-resolution MWIR images are reconstructed at (**a**) 0.125, (**b**) 0.25, (**c**) 0.375 and (**d**) 0.5. A small area of each image is zoomed in to highlight the BSA.

If high-resolution images are reconstructed from these low-resolution MWIR images, in which aperture interference phenomenon is not obvious, BSA can be alleviated. In Figure 8, the reconstructed high-resolution images with compression ratios of 0.125, 0.25, 0.375 and 0.5 are shown, and the same areas are zoomed in to highlight the BSA.

**Figure 9:**
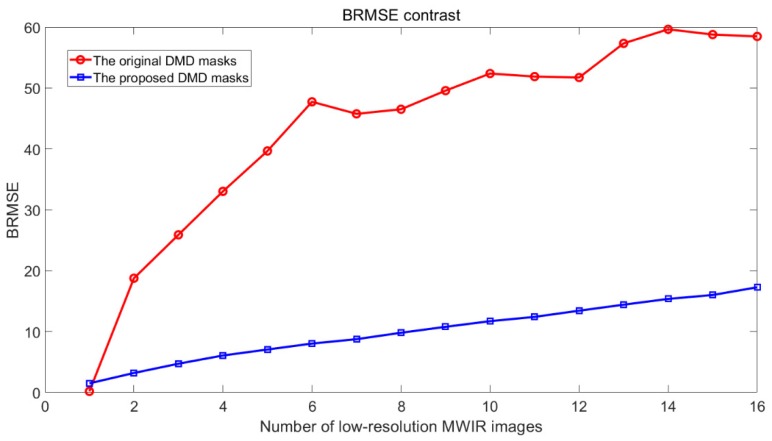
The contrast of BRMSE between the two DMD mask design strategies.

To objectively describe the effect of the proposed DMD masks, we use BRMSE to evaluate the BSA of the reconstructed high-resolution images. With the two strategies of DMD mask design, one is the original [19] and the other is the proposed, high-resolution MWIR images, using the blackbody as imaged scene, reconstructed with different amounts of low-resolution images, from 1 to 16. For our MWIR FPA CI system, 16 is the maximum sampling number, equal to the number of micro-mirrors in one block. The contrast of BRMSE for the two DMD mask design strategies is shown in Figure 9. It is obvious that the proposed DMD masks can effectively reduce the BRMSE of the reconstructed high-resolution images, which means the BSA caused by aperture interference in MWIR FPA CI is effectively suppressed.

**Figure 10:**
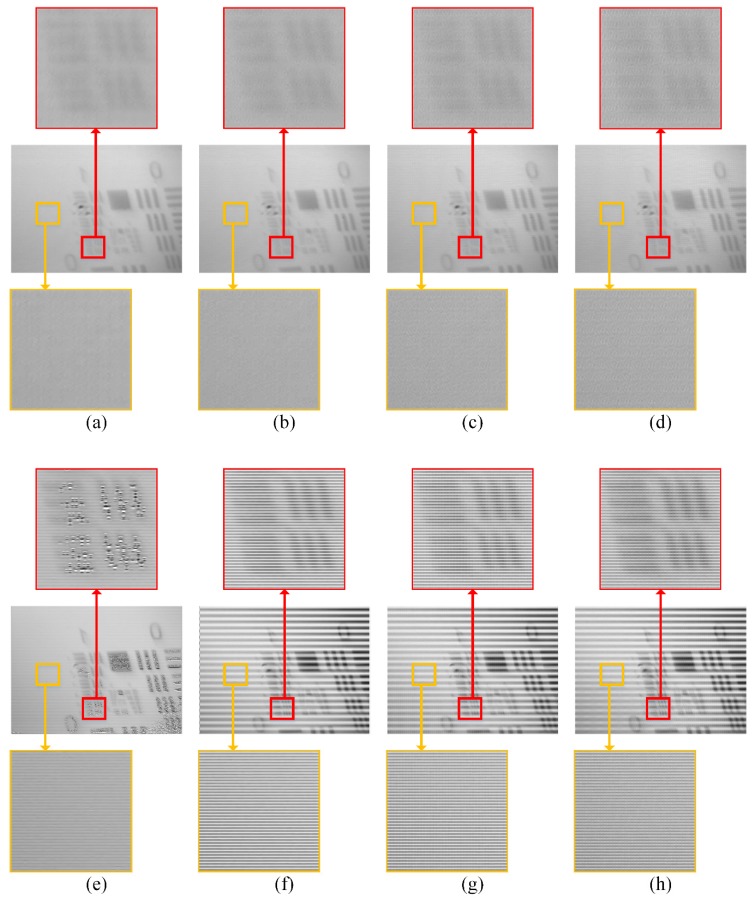
The high-resolution MWIR images are reconstructed with (**a**–**d**) the proposed DMD masks and (**e**–**h**) the original DMD masks. In each row, from left to right, the compression ratios are 0.125, 0.25, 0.375 and 0.5 respectively.

Using a transmission type of USAF resolution test chart as imaged scene, low-resolution MWIR images are obtained with the two strategies of DMD mask design. Then, the reconstructed high-resolution images are computed with compression ratios of 0.125, 0.25, 0.375 and 0.5. To highlight the BSA in the reconstructed images, some areas are zoomed in, which are shown in Figure 10.

In the first row, images are reconstructed using the low-resolution MWIR images obtained with the proposed DMD masks, at compression ratios of 0.125, 0.25, 0.375 and 0.5. In the second row, images are reconstructed using the images obtained with the original DMD masks. In the flat zoomed-in zones, which are highlighted by yellow borders, the proposed DMD masks are obviously superior to the original ones. For the textured zoomed-in zones, which are highlighted by red borders, the image quality of the original DMD masks is significantly reduced in Figure 10e, but looks sharper in f–h than a–d, which were obtained using the proposed DMD masks. The reason for this phenomenon is, for the original DMD masks, due to the BSA, the gray values are unevenly assigned to every two adjacent row, making one row bright and the other dark. In viewing the overall image, the image may appear shaper, but the local features of the image are seriously damaged, which is a great obstacle for traditional digital image processing algorithms [35]. However, for the proposed DMD masks, the local features of the image are better preserved, and the line pairs are not cut into pieces as with the original DMD masks. This improvement is helpful for automatic target recognition [36]. Although the BSA cannot be completely eliminated, image qualities are improved by the proposed DMD masks in comparison to the original ones.

## 5. Conclusions

In MWIR FPA CI, aperture interference phenomena are inevitable, which aggravate the BSA in the reconstructed high-resolution images. The quality of the reconstructed images is getting worse as the compression ratio increases. As a result, the aperture interference reduces the practicality of the MWIR FPA CI system. In this paper, we analyze the relationship between the aperture interference and BSA, and propose a novel strategy of DMD mask design, which can effectively suppress the BSA. Compared with a simple application of the Hadamard matrix, the proposed DMD masks that can alleviate the BSA of the high-resolution images reconstructed with MWIR, at the cost of a little more storage space and computation cost. Compared with random binary codes, the proposed DMD masks can significantly reduce storage space and computation cost, and maximize the reconstruction efficiency, as the patterns for each block are completely distinct between the DMD masks. To objectively evaluate the BSA in FPA CI, we propose a new evaluation index, BRMSE. With the actual MWIR FPA CI system, we demonstrate that the proposed DMD masks can significantly reduce the BRMSE, which means the BSA is effectively suppressed. The proposed DMD mask design strategy improves the practicability of MWIR FPA CI.

In FPA CI, aperture interference is closely related to the imaging wavelength. As another available waveband, long wave infrared (LWIR) is also important for infrared imaging and has a high research value. In future work, we will investigate the aperture interference in LWIR FPA CI.
